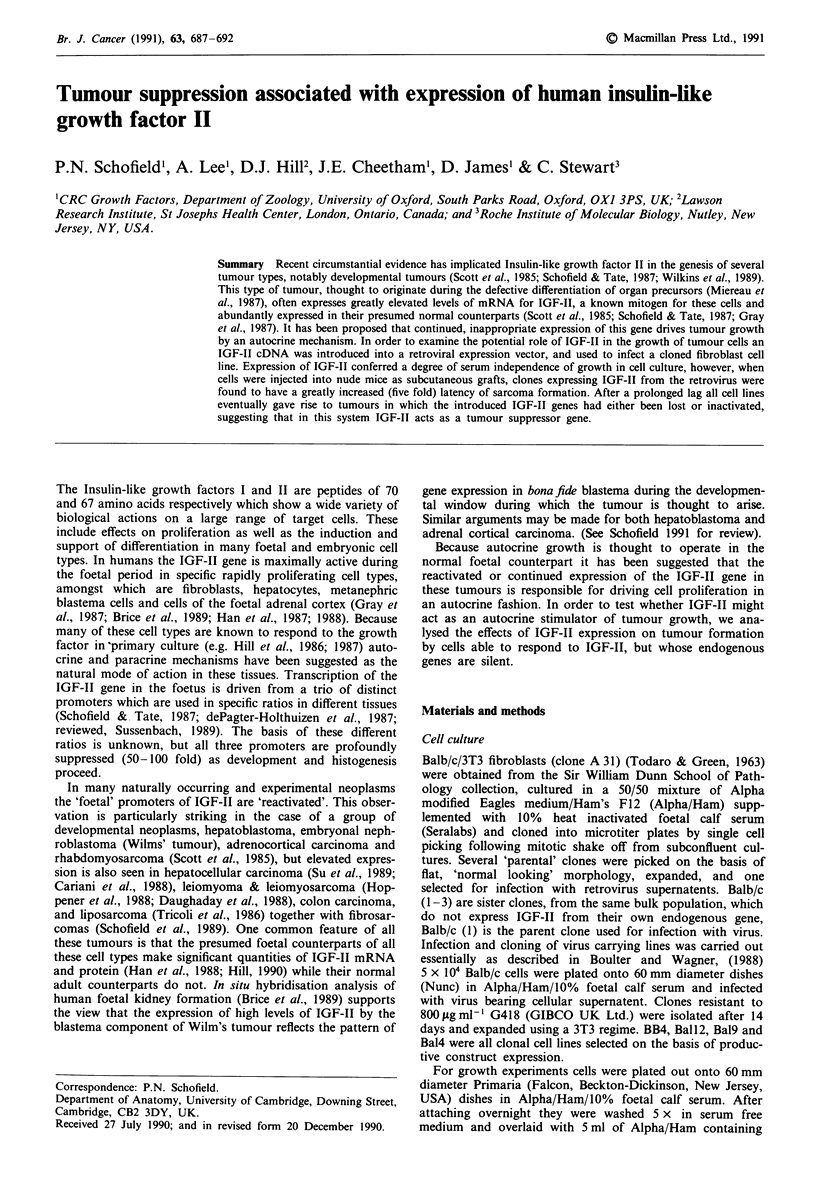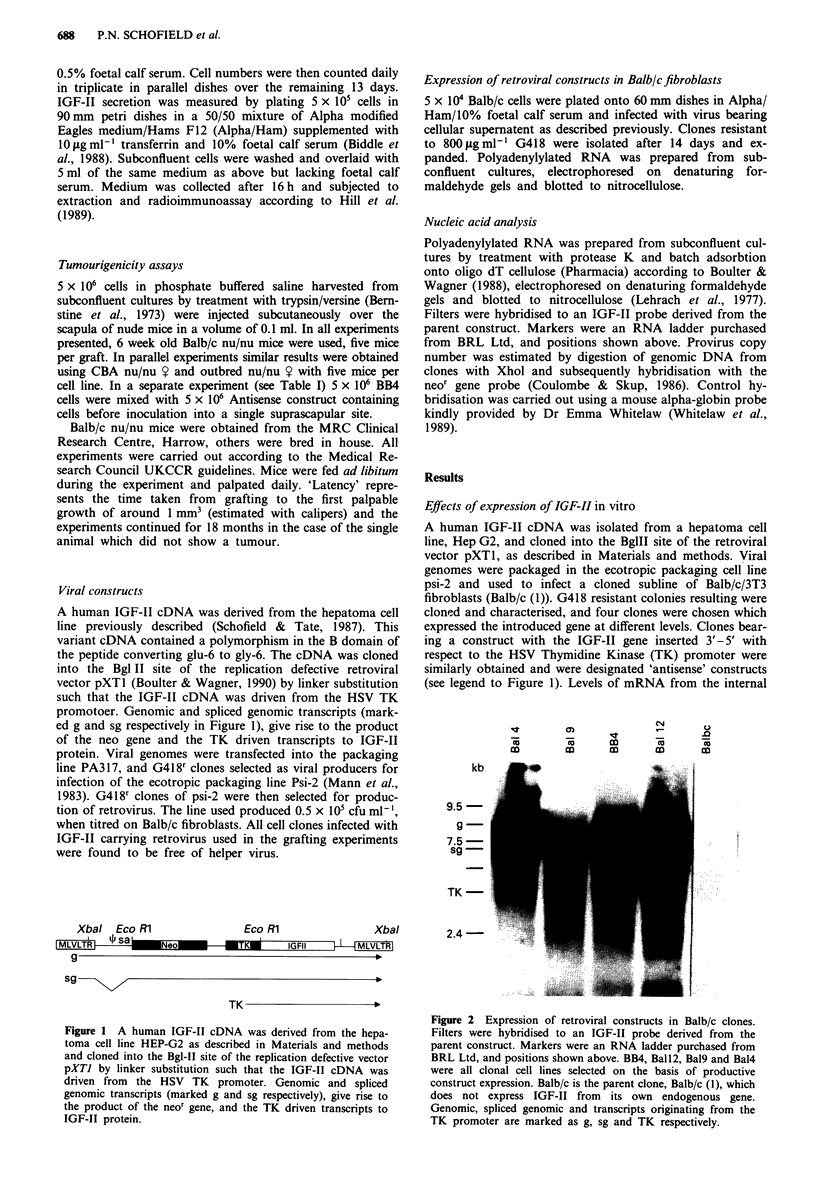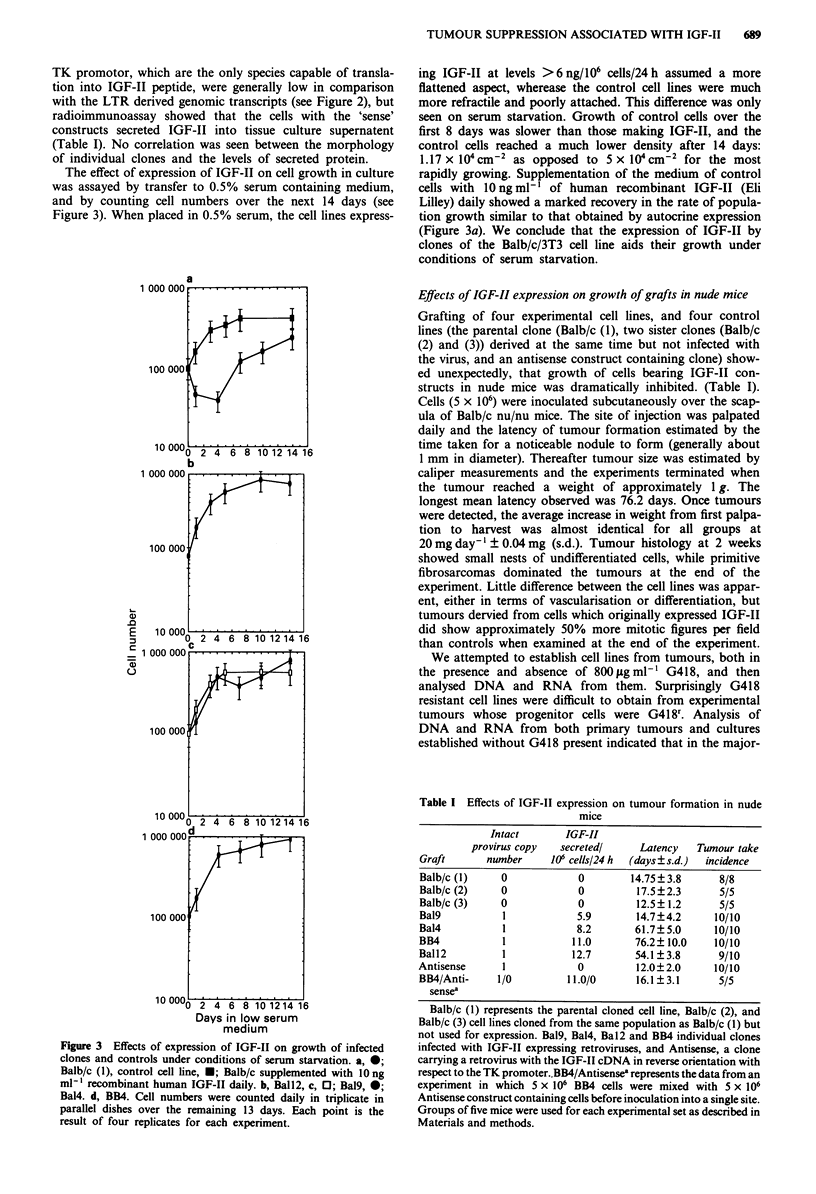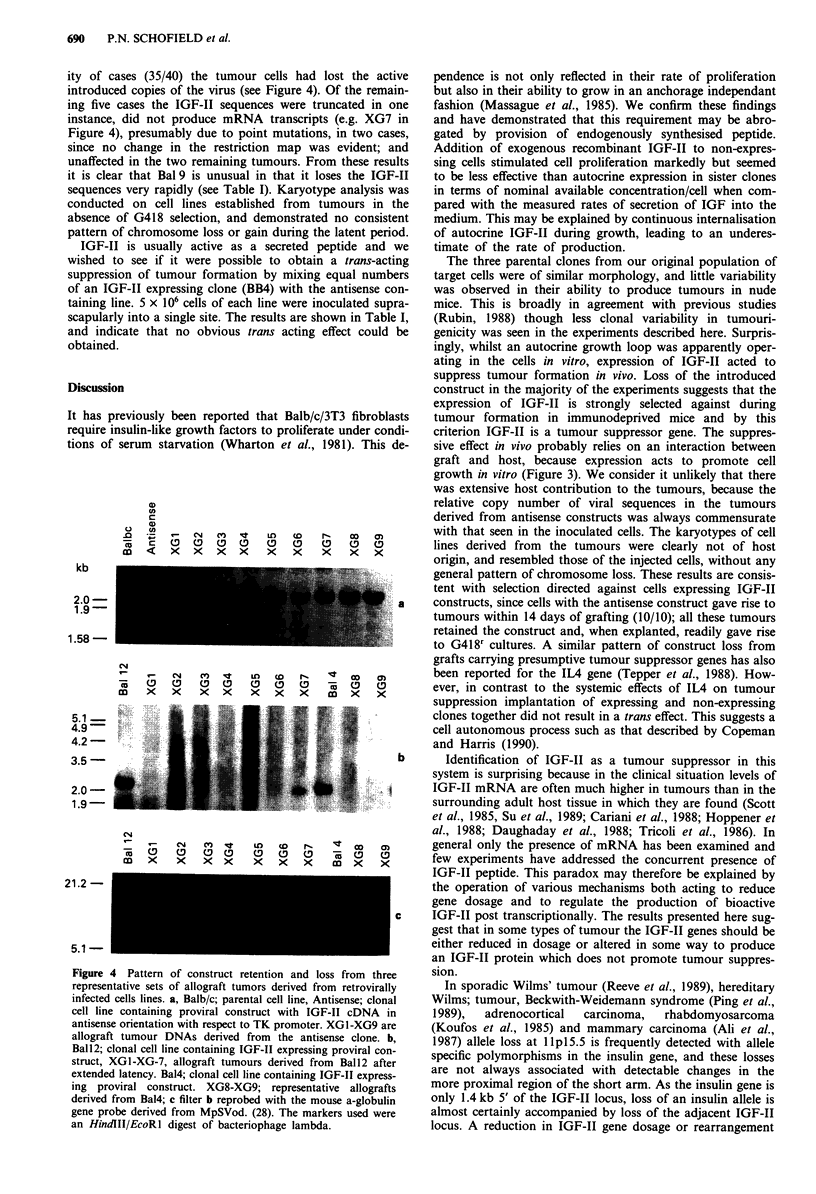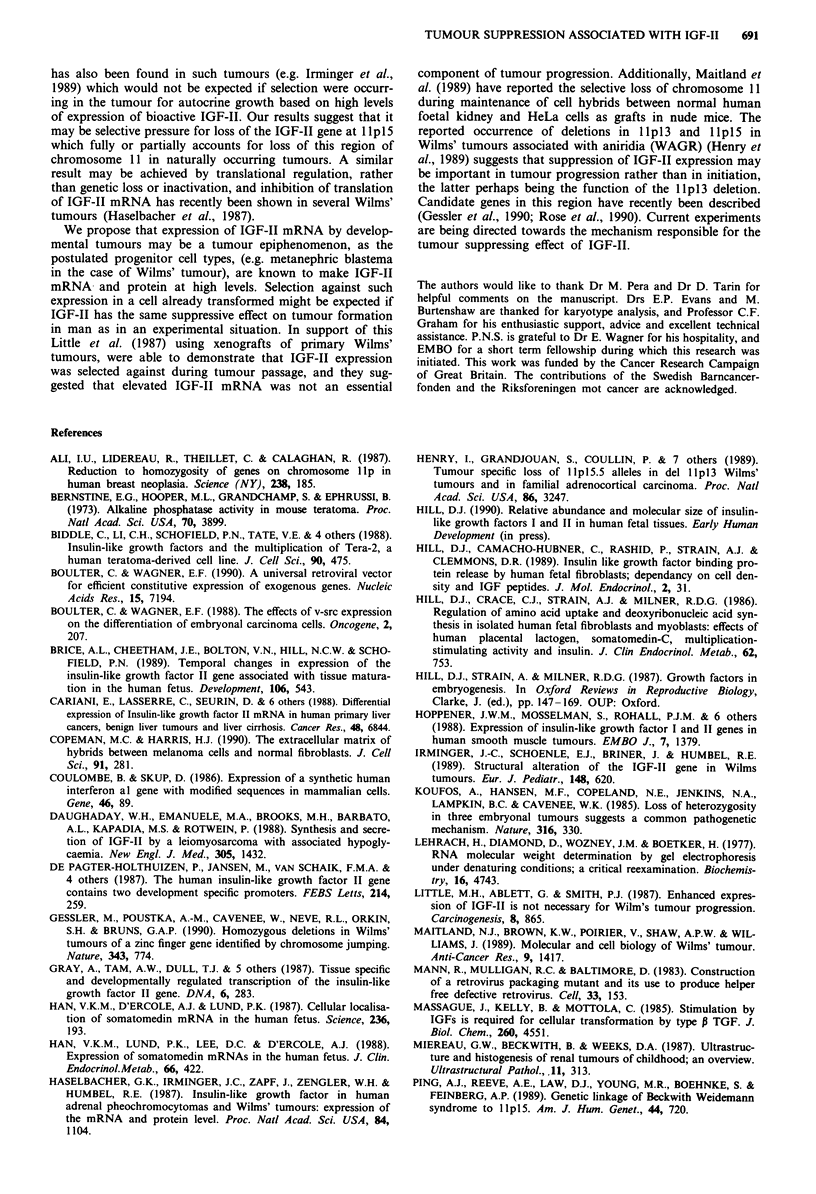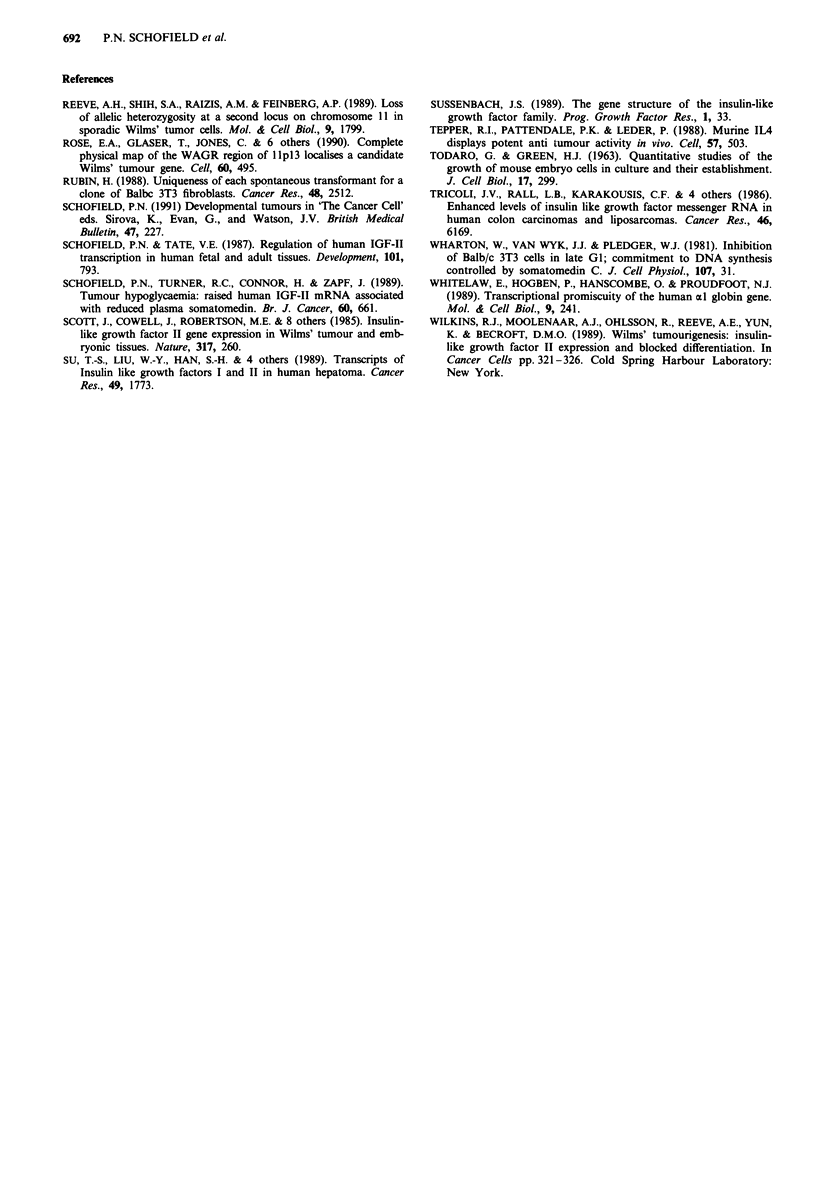# Tumour suppression associated with expression of human insulin-like growth factor II.

**DOI:** 10.1038/bjc.1991.156

**Published:** 1991-05

**Authors:** P. N. Schofield, A. Lee, D. J. Hill, J. E. Cheetham, D. James, C. Stewart

**Affiliations:** Department of Zoology, University of Oxford, UK.

## Abstract

**Images:**


*Br. J. Cancer* (1991), **63**, 687–692 © Macmillan Press Ltd., 1991

### Tumour suppression associated with expression of human insulin-like growth factor II

P.N. Schofield[1], A. Lee[1], D.J. Hill[2], J.E. Cheetham[1], D. James[1] & C. Stewart[3]

[1]*CRC Growth Factors, Department of Zoology, University of Oxford, South Parks Road, Oxford, OX1 3PS, UK;* [2]*Lawson Research Institute, St Josephs Health Center, London, Ontario, Canada; and* [3]*Roche Institute of Molecular Biology, Nutley, New Jersey, NY, USA.*

**Summary** Recent circumstantial evidence has implicated Insulin-like growth factor II in the genesis of several tumour types, notably developmental tumours (Scott *et al*., 1985; Schofield & Tate, 1987; Wilkins *et al*., 1989). This type of tumour, thought to originate during the defective differentiation of organ precursors (Miereau *et al*., 1987), often expresses greatly elevated levels of mRNA for IGF-II, a known mitogen for these cells and abundantly expressed in their presumed normal counterparts (Scott *et al*., 1985; Schofield & Tate, 1987; Gray *et al*., 1987). It has been proposed that continued, inappropriate expression of this gene drives tumour growth by an autocrine mechanism. In order to examine the potential role of IGF-II in the growth of tumour cells an IGF-II cDNA was introduced into a retroviral expression vector, and used to infect a cloned fibroblast cell line. Expression of IGF-II conferred a degree of serum independence of growth in cell culture, however, when cells were injected into nude mice as subcutaneous grafts, clones expressing IGF-II from the retrovirus were found to have a greatly increased (five fold) latency of sarcoma formation. After a prolonged lag all cell lines eventually gave rise to tumours in which the introduced IGF-II genes had either been lost or inactivated, suggesting that in this system IGF-II acts as a tumour suppressor gene.

The Insulin-like growth factors I and II are peptides of 70 and 67 amino acids respectively which show a wide variety of biological actions on a large range of target cells. These include effects on proliferation as well as the induction and support of differentiation in many foetal and embryonic cell types. In humans the IGF-II gene is maximally active during the foetal period in specific rapidly proliferating cell types, amongst which are fibroblasts, hepatocytes, metanephric blastema cells and cells of the foetal adrenal cortex (Gray *et al*., 1987; Brice *et al*., 1989; Han *et al*., 1987; 1988). Because many of these cell types are known to respond to the growth factor in primary culture (e.g. Hill *et al*., 1986; 1987) autocrine and paracrine mechanisms have been suggested as the natural mode of action in these tissues. Transcription of the IGF-II gene in the foetus is driven from a trio of distinct promoters which are used in specific ratios in different tissues (Schofield & Tate, 1987; dePagter-Holthuizen *et al*., 1987; reviewed, Sussenbach, 1989). The basis of these different ratios is unknown, but all three promoters are profoundly suppressed (50–100 fold) as development and histogenesis proceed.

In many naturally occurring and experimental neoplasms the 'foetal' promoters of IGF-II are 'reactivated'. This observation is particularly striking in the case of a group of developmental neoplasms, hepatoblastoma, embryonal nephroblastoma (Wilms' tumour), adrenocortical carcinoma and rhabdomyosarcoma (Scott *et al*., 1985), but elevated expression is also seen in hepatocellular carcinoma (Su *et al*., 1989; Cariani *et al*., 1988), leiomyoma & leiomyosarcoma (Hoppener *et al*., 1988; Daughaday *et al*., 1988), colon carcinoma, and liposarcoma (Tricoli *et al*., 1986) together with fibrosarcomas (Schofield *et al*., 1989). One common feature of all these tumours is that the presumed foetal counterparts of all these cell types make significant quantities of IGF-II mRNA and protein (Han *et al*., 1988; Hill, 1990) while their normal adult counterparts do not. *In situ* hybridisation analysis of human foetal kidney formation (Brice *et al*., 1989) supports the view that the expression of high levels of IGF-II by the blastema component of Wilm's tumour reflects the pattern of gene expression in *bona fide* blastema during the developmental window during which the tumour is thought to arise. Similar arguments may be made for both hepatoblastoma and adrenal cortical carcinoma. (See Schofield 1991 for review).

Because autocrine growth is thought to operate in the normal foetal counterpart it has been suggested that the reactivated or continued expression of the IGF-II gene in these tumours is responsible for driving cell proliferation in an autocrine fashion. In order to test whether IGF-II might act as an autocrine stimulator of tumour growth, we analysed the effects of IGF-II expression on tumour formation by cells able to respond to IGF-II, but whose endogenous genes are silent.

### Materials and methods

### *Cell culture*

Balb/c/3T3 fibroblasts (clone A 31) (Todaro & Green, 1963) were obtained from the Sir William Dunn School of Pathology collection, cultured in a 50/50 mixture of Alpha modified Eagles medium/Ham's F12 (Alpha/Ham) supplemented with 10% heat inactivated foetal calf serum (Seralabs) and cloned into microtiter plates by single cell picking following mitotic shake off from subconfluent cultures. Several 'parental' clones were picked on the basis of flat, 'normal looking' morphology, expanded, and one selected for infection with retrovirus supernatents. Balb/c (1–3) are sister clones, from the same bulk population, which do not express IGF-II from their own endogenous gene, Balb/c (1) is the parent clone used for infection with virus. Infection and cloning of virus carrying lines was carried out essentially as described in Boulter and Wagner, (1988) $5 \times 10^4$ Balb/c cells were plated onto 60 mm diameter dishes (Nunc) in Alpha/Ham/10% foetal calf serum and infected with virus bearing cellular supernatent. Clones resistant to 800 $\mu g\, ml^{-1}$ G418 (GIBCO UK Ltd.) were isolated after 14 days and expanded using a 3T3 regime. BB4, Bal12, Bal9 and Bal4 were all clonal cell lines selected on the basis of productive construct expression.

For growth experiments cells were plated out onto 60 mm diameter Primaria (Falcon, Beckton-Dickinson, New Jersey, USA) dishes in Alpha/Ham/10% foetal calf serum. After attaching overnight they were washed 5× in serum free medium and overlaid with 5 ml of Alpha/Ham containing

Correspondence: P.N. Schofield.
Department of Anatomy, University of Cambridge, Downing Street, Cambridge, CB2 3DY, UK.
Received 27 July 1990; and in revised form 20 December 1990.

0.5% foetal calf serum. Cell numbers were then counted daily in triplicate in parallel dishes over the remaining 13 days. IGF-II secretion was measured by plating $5 \times 10^5$ cells in 90 mm petri dishes in a 50/50 mixture of Alpha modified Eagles medium/Hams F12 (Alpha/Ham) supplemented with 10 $\mu g\, ml^{-1}$ transferrin and 10% foetal calf serum (Biddle *et al.*, 1988). Subconfluent cells were washed and overlaid with 5 ml of the same medium as above but lacking foetal calf serum. Medium was collected after 16 h and subjected to extraction and radioimmunoassay according to Hill *et al.* (1989).

*Tumourigenicity assays*

$5 \times 10^6$ cells in phosphate buffered saline harvested from subconfluent cultures by treatment with trypsin/versine (Bernstine *et al.*, 1973) were injected subcutaneously over the scapula of nude mice in a volume of 0.1 ml. In all experiments presented, 6 week old Balb/c nu/nu mice were used, five mice per graft. In parallel experiments similar results were obtained using CBA nu/nu ♀ and outbred nu/nu ♀ with five mice per cell line. In a separate experiment (see Table I) $5 \times 10^6$ BB4 cells were mixed with $5 \times 10^6$ Antisense construct containing cells before inoculation into a single suprascapular site.

Balb/c nu/nu mice were obtained from the MRC Clinical Research Centre, Harrow, others were bred in house. All experiments were carried out according to the Medical Research Council UKCCR guidelines. Mice were fed *ad libitum* during the experiment and palpated daily. 'Latency' represents the time taken from grafting to the first palpable growth of around 1 $mm^3$ (estimated with calipers) and the experiments continued for 18 months in the case of the single animal which did not show a tumour.

*Viral constructs*

A human IGF-II cDNA was derived from the hepatoma cell line previously described (Schofield & Tate, 1987). This variant cDNA contained a polymorphism in the B domain of the peptide converting glu-6 to gly-6. The cDNA was cloned into the Bgl II site of the replication defective retroviral vector pXT1 (Boulter & Wagner, 1990) by linker substitution such that the IGF-II cDNA was driven from the HSV TK promotoer. Genomic and spliced genomic transcripts (marked g and sg respectively in Figure 1), give rise to the product of the neo gene and the TK driven transcripts to IGF-II protein. Viral genomes were transfected into the packaging line PA317, and G418$^r$ clones selected as viral producers for infection of the ecotropic packaging line Psi-2 (Mann *et al.*, 1983). G418$^r$ clones of psi-2 were then selected for production of retrovirus. The line used produced $0.5 \times 10^5$ cfu $ml^{-1}$, when titred on Balb/c fibroblasts. All cell clones infected with IGF-II carrying retrovirus used in the grafting experiments were found to be free of helper virus.

**Figure 1** A human IGF-II cDNA was derived from the hepatoma cell line HEP-G2 as described in Materials and methods and cloned into the Bgl-II site of the replication defective vector p*XT1* by linker substitution such that the IGF-II cDNA was driven from the HSV TK promoter. Genomic and spliced genomic transcripts (marked g and sg respectively), give rise to the product of the neo$^r$ gene, and the TK driven transcripts to IGF-II protein.

*Expression of retroviral constructs in Balb/c fibroblasts*

$5 \times 10^4$ Balb/c cells were plated onto 60 mm dishes in Alpha/Ham/10% foetal calf serum and infected with virus bearing cellular supernatent as described previously. Clones resistant to 800 $\mu g\, ml^{-1}$ G418 were isolated after 14 days and expanded. Polyadenylylated RNA was prepared from subconfluent cultures, electrophoresed on denaturing formaldehyde gels and blotted to nitrocellulose.

*Nucleic acid analysis*

Polyadenylylated RNA was prepared from subconfluent cultures by treatment with protease K and batch adsorbtion onto oligo dT cellulose (Pharmacia) according to Boulter & Wagner (1988), electrophoresed on denaturing formaldehyde gels and blotted to nitrocellulose (Lehrach *et al.*, 1977). Filters were hybridised to an IGF-II probe derived from the parent construct. Markers were an RNA ladder purchased from BRL Ltd, and positions shown above. Provirus copy number was estimated by digestion of genomic DNA from clones with XhoI and subsequently hybridisation with the neo$^r$ gene probe (Coulombe & Skup, 1986). Control hybridisation was carried out using a mouse alpha-globin probe kindly provided by Dr Emma Whitelaw (Whitelaw *et al.*, 1989).

### Results

*Effects of expression of IGF-II* in vitro

A human IGF-II cDNA was isolated from a hepatoma cell line, Hep G2, and cloned into the BglII site of the retroviral vector pXT1, as described in Materials and methods. Viral genomes were packaged in the ecotropic packaging cell line psi-2 and used to infect a cloned subline of Balb/c/3T3 fibroblasts (Balb/c (1)). G418 resistant colonies resulting were cloned and characterised, and four clones were chosen which expressed the introduced gene at different levels. Clones bearing a construct with the IGF-II gene inserted 3′–5′ with respect to the HSV Thymidine Kinase (TK) promoter were similarly obtained and were designated 'antisense' constructs (see legend to Figure 1). Levels of mRNA from the internal

**Figure 2** Expression of retroviral constructs in Balb/c clones. Filters were hybridised to an IGF-II probe derived from the parent construct. Markers were an RNA ladder purchased from BRL Ltd, and positions shown above. BB4, Bal12, Bal9 and Bal4 were all clonal cell lines selected on the basis of productive construct expression. Balb/c is the parent clone, Balb/c (1), which does not express IGF-II from its own endogenous gene. Genomic, spliced genomic and transcripts originating from the TK promoter are marked as g, sg and TK respectively.

TK promotor, which are the only species capable of translation into IGF-II peptide, were generally low in comparison with the LTR derived genomic transcripts (see Figure 2), but radioimmunoassay showed that the cells with the 'sense' constructs secreted IGF-II into tissue culture supernatent (Table I). No correlation was seen between the morphology of individual clones and the levels of secreted protein.

The effect of expression of IGF-II on cell growth in culture was assayed by transfer to 0.5% serum containing medium, and by counting cell numbers over the next 14 days (see Figure 3). When placed in 0.5% serum, the cell lines expressing IGF-II at levels $>6\,ng/10^6$ cells/24 h assumed a more flattened aspect, wherease the control cell lines were much more refractile and poorly attached. This difference was only seen on serum starvation. Growth of control cells over the first 8 days was slower than those making IGF-II, and the control cells reached a much lower density after 14 days: $1.17 \times 10^4\,cm^{-2}$ as opposed to $5 \times 10^4\,cm^{-2}$ for the most rapidly growing. Supplementation of the medium of control cells with $10\,ng\,ml^{-1}$ of human recombinant IGF-II (Eli Lilley) daily showed a marked recovery in the rate of population growth similar to that obtained by autocrine expression (Figure 3*a*). We conclude that the expression of IGF-II by clones of the Balb/c/3T3 cell line aids their growth under conditions of serum starvation.

**Figure 3** Effects of expression of IGF-II on growth of infected clones and controls under conditions of serum starvation. **a**, ●; Balb/c (1), control cell line, ■; Balb/c supplemented with 10 ng $ml^{-1}$ recombinant human IGF-II daily. **b**, Bal12, **c**, □; Bal9, ●; Bal4. **d**, BB4. Cell numbers were counted daily in triplicate in parallel dishes over the remaining 13 days. Each point is the result of four replicates for each experiment.

### *Effects of IGF-II expression on growth of grafts in nude mice*

Grafting of four experimental cell lines, and four control lines (the parental clone (Balb/c (1), two sister clones (Balb/c (2) and (3)) derived at the same time but not infected with the virus, and an antisense construct containing clone) showed unexpectedly, that growth of cells bearing IGF-II constructs in nude mice was dramatically inhibited. (Table I). Cells ($5 \times 10^6$) were inoculated subcutaneously over the scapula of Balb/c nu/nu mice. The site of injection was palpated daily and the latency of tumour formation estimated by the time taken for a noticeable nodule to form (generally about 1 mm in diameter). Thereafter tumour size was estimated by caliper measurements and the experiments terminated when the tumour reached a weight of approximately 1 *g*. The longest mean latency observed was 76.2 days. Once tumours were detected, the average increase in weight from first palpation to harvest was almost identical for all groups at $20\,mg\,day^{-1} \pm 0.04\,mg$ (s.d.). Tumour histology at 2 weeks showed small nests of undifferentiated cells, while primitive fibrosarcomas dominated the tumours at the end of the experiment. Little difference between the cell lines was apparent, either in terms of vascularisation or differentiation, but tumours dervied from cells which originally expressed IGF-II did show approximately 50% more mitotic figures per field than controls when examined at the end of the experiment.

We attempted to establish cell lines from tumours, both in the presence and absence of $800\,\mu g\,ml^{-1}$ G418, and then analysed DNA and RNA from them. Surprisingly G418 resistant cell lines were difficult to obtain from experimental tumours whose progenitor cells were G418$^r$. Analysis of DNA and RNA from both primary tumours and cultures established without G418 present indicated that in the major-

**Table I** Effects of IGF-II expression on tumour formation in nude mice

| *Graft* | *Intact provirus copy number* | *IGF-II secreted/ $10^6$ cells/24 h* | *Latency (days ± s.d.)* | *Tumour take incidence* |
|---|---|---|---|---|
| Balb/c (1) | 0 | 0 | 14.75±3.8 | 8/8 |
| Balb/c (2) | 0 | 0 | 17.5±2.3 | 5/5 |
| Balb/c (3) | 0 | 0 | 12.5±1.2 | 5/5 |
| Bal9 | 1 | 5.9 | 14.7±4.2 | 10/10 |
| Bal4 | 1 | 8.2 | 61.7±5.0 | 10/10 |
| BB4 | 1 | 11.0 | 76.2±10.0 | 10/10 |
| Bal12 | 1 | 12.7 | 54.1±3.8 | 9/10 |
| Antisense | 1 | 0 | 12.0±2.0 | 10/10 |
| BB4/Antisense[a] | 1/0 | 11.0/0 | 16.1±3.1 | 5/5 |

Balb/c (1) represents the parental cloned cell line, Balb/c (2), and Balb/c (3) cell lines cloned from the same population as Balb/c (1) but not used for expression. Bal9, Bal4, Bal2 and BB4 individual clones infected with IGF-II expressing retroviruses, and Antisense, a clone carrying a retrovirus with the IGF-II cDNA in reverse orientation with respect to the TK promoter..BB4/Antisense[a] represents the data from an experiment in which $5 \times 10^6$ BB4 cells were mixed with $5 \times 10^6$ Antisense construct containing cells before inoculation into a single site. Groups of five mice were used for each experimental set as described in Materials and methods.

ity of cases (35/40) the tumour cells had lost the active introduced copies of the virus (see Figure 4). Of the remaining five cases the IGF-II sequences were truncated in one instance, did not produce mRNA transcripts (e.g. XG7 in Figure 4), presumably due to point mutations, in two cases, since no change in the restriction map was evident; and unaffected in the two remaining tumours. From these results it is clear that Bal 9 is unusual in that it loses the IGF-II sequences very rapidly (see Table I). Karyotype analysis was conducted on cell lines established from tumours in the absence of G418 selection, and demonstrated no consistent pattern of chromosome loss or gain during the latent period.

IGF-II is usually active as a secreted peptide and we wished to see if it were possible to obtain a *trans*-acting suppression of tumour formation by mixing equal numbers of an IGF-II expressing clone (BB4) with the antisense containing line. $5 \times 10^6$ cells of each line were inoculated suprascapularly into a single site. The results are shown in Table I, and indicate that no obvious *trans* acting effect could be obtained.

### Discussion

It has previously been reported that Balb/c/3T3 fibroblasts require insulin-like growth factors to proliferate under conditions of serum starvation (Wharton *et al.*, 1981). This dependence is not only reflected in their rate of proliferation but also in their ability to grow in an anchorage independant fashion (Massague *et al.*, 1985). We confirm these findings and have demonstrated that this requirement may be abrogated by provision of endogenously synthesised peptide. Addition of exogenous recombinant IGF-II to non-expressing cells stimulated cell proliferation markedly but seemed to be less effective than autocrine expression in sister clones in terms of nominal available concentration/cell when compared with the measured rates of secretion of IGF into the medium. This may be explained by continuous internalisation of autocrine IGF-II during growth, leading to an underestimate of the rate of production.

**Figure 4** Pattern of construct retention and loss from three representative sets of allograft tumors derived from retrovirally infected cells lines. **a**, Balb/c; parental cell line, Antisense; clonal cell line containing proviral construct with IGF-II cDNA in antisense orientation with respect to TK promoter. XG1-XG9 are allograft tumour DNAs derived from the antisense clone. **b**, Bal12; clonal cell line containing IGF-II expressing proviral construct, XG1-XG-7, allograft tumours derived from Bal12 after extended latency. Bal4; clonal cell line containing IGF-II expressing proviral construct. XG8-XG9; representative allografts derived from Bal4; **c** filter **b** reprobed with the mouse a-globulin gene probe derived from MpSVod. (28). The markers used were an *Hind*III/*Eco*R1 digest of bacteriophage lambda.

The three parental clones from our original population of target cells were of similar morphology, and little variability was observed in their ability to produce tumours in nude mice. This is broadly in agreement with previous studies (Rubin, 1988) though less clonal variability in tumourigenicity was seen in the experiments described here. Surprisingly, whilst an autocrine growth loop was apparently operating in the cells *in vitro*, expression of IGF-II acted to suppress tumour formation *in vivo*. Loss of the introduced construct in the majority of the experiments suggests that the expression of IGF-II is strongly selected against during tumour formation in immunodeprived mice and by this criterion IGF-II is a tumour suppressor gene. The suppressive effect *in vivo* probably relies on an interaction between graft and host, because expression acts to promote cell growth *in vitro* (Figure 3). We consider it unlikely that there was extensive host contribution to the tumours, because the relative copy number of viral sequences in the tumours derived from antisense constructs was always commensurate with that seen in the inoculated cells. The karyotypes of cell lines derived from the tumours were clearly not of host origin, and resembled those of the injected cells, without any general pattern of chromosome loss. These results are consistent with selection directed against cells expressing IGF-II constructs, since cells with the antisense construct gave rise to tumours within 14 days of grafting (10/10); all these tumours retained the construct and, when explanted, readily gave rise to G418$^r$ cultures. A similar pattern of construct loss from grafts carrying presumptive tumour suppressor genes has also been reported for the IL4 gene (Tepper *et al.*, 1988). However, in contrast to the systemic effects of IL4 on tumour suppression implantation of expressing and non-expressing clones together did not result in a *trans* effect. This suggests a cell autonomous process such as that described by Copeman and Harris (1990).

Identification of IGF-II as a tumour suppressor in this system is surprising because in the clinical situation levels of IGF-II mRNA are often much higher in tumours than in the surrounding adult host tissue in which they are found (Scott *et al.*, 1985, Su *et al.*, 1989; Cariani *et al.*, 1988; Hoppener *et al.*, 1988; Daughaday *et al.*, 1988; Tricoli *et al.*, 1986). In general only the presence of mRNA has been examined and few experiments have addressed the concurrent presence of IGF-II peptide. This paradox may therefore be explained by the operation of various mechanisms both acting to reduce gene dosage and to regulate the production of bioactive IGF-II post transcriptionally. The results presented here suggest that in some types of tumour the IGF-II genes should be either reduced in dosage or altered in some way to produce an IGF-II protein which does not promote tumour suppression.

In sporadic Wilms' tumour (Reeve *et al.*, 1989), hereditary Wilms; tumour, Beckwith-Weidemann syndrome (Ping *et al.*, 1989), adrenocortical carcinoma, rhabdomyosarcoma (Koufos *et al.*, 1985) and mammary carcinoma (Ali *et al.*, 1987) allele loss at 11p15.5 is frequently detected with allele specific polymorphisms in the insulin gene, and these losses are not always associated with detectable changes in the more proximal region of the short arm. As the insulin gene is only 1.4 kb 5′ of the IGF-II locus, loss of an insulin allele is almost certainly accompanied by loss of the adjacent IGF-II locus. A reduction in IGF-II gene dosage or rearrangement has also been found in such tumours (e.g. Irminger *et al.*, 1989) which would not be expected if selection were occurring in the tumour for autocrine growth based on high levels of expression of bioactive IGF-II. Our results suggest that it may be selective pressure for loss of the IGF-II gene at 11p15 which fully or partially accounts for loss of this region of chromosome 11 in naturally occurring tumours. A similar result may be achieved by translational regulation, rather than genetic loss or inactivation, and inhibition of translation of IGF-II mRNA has recently been shown in several Wilms' tumours (Haselbacher *et al.*, 1987).

We propose that expression of IGF-II mRNA by developmental tumours may be a tumour epiphenomenon, as the postulated progenitor cell types, (e.g. metanephric blastema in the case of Wilms' tumour), are known to make IGF-II mRNA and protein at high levels. Selection against such expression in a cell already transformed might be expected if IGF-II has the same suppressive effect on tumour formation in man as in an experimental situation. In support of this Little *et al.* (1987) using xenografts of primary Wilms' tumours, were able to demonstrate that IGF-II expression was selected against during tumour passage, and they suggested that elevated IGF-II mRNA was not an essential component of tumour progression. Additionally, Maitland *et al.* (1989) have reported the selective loss of chromosome 11 during maintenance of cell hybrids between normal human foetal kidney and HeLa cells as grafts in nude mice. The reported occurrence of deletions in 11p13 and 11p15 in Wilms' tumours associated with aniridia (WAGR) (Henry *et al.*, 1989) suggests that suppression of IGF-II expression may be important in tumour progression rather than in initiation, the latter perhaps being the function of the 11p13 deletion. Candidate genes in this region have recently been described (Gessler *et al.*, 1990; Rose *et al.*, 1990). Current experiments are being directed towards the mechanism responsible for the tumour suppressing effect of IGF-II.

The authors would like to thank Dr M. Pera and Dr D. Tarin for helpful comments on the manuscript. Drs E.P. Evans and M. Burtenshaw are thanked for karyotype analysis, and Professor C.F. Graham for his enthusiastic support, advice and excellent technical assistance. P.N.S. is grateful to Dr E. Wagner for his hospitality, and EMBO for a short term fellowship during which this research was initiated. This work was funded by the Cancer Research Campaign of Great Britain. The contributions of the Swedish Barncancerfonden and the Riksforeningen mot cancer are acknowledged.

### References

ALI, I.U., LIDEREAU, R., THEILLET, C. & CALAGHAN, R. (1987). Reduction to homozygosity of genes on chromosome 11p in human breast neoplasia. *Science (NY)*, **238**, 185.

BERNSTINE, E.G., HOOPER, M.L., GRANDCHAMP, S. & EPHRUSSI, B. (1973). Alkaline phosphatase activity in mouse teratoma. *Proc. Natl Acad. Sci. USA*, **70**, 3899.

BIDDLE, C., LI, C.H., SCHOFIELD, P.N., TATE, V.E. & 4 others (1988). Insulin-like growth factors and the multiplication of Tera-2, a human teratoma-derived cell line. *J. Cell Sci.*, **90**, 475.

BOULTER, C. & WAGNER, E.F. (1990). A universal retroviral vector for efficient constitutive expression of exogenous genes. *Nucleic Acids Res.*, **15**, 7194.

BOULTER, C. & WAGNER, E.F. (1988). The effects of v-src expression on the differentiation of embryonal carcinoma cells. *Oncogene*, **2**, 207.

BRICE, A.L., CHEETHAM, J.E., BOLTON, V.N., HILL, N.C.W. & SCHOFIELD, P.N. (1989). Temporal changes in expression of the insulin-like growth factor II gene associated with tissue maturation in the human fetus. *Development*, **106**, 543.

CARIANI, E., LASSERRE, C., SEURIN, D. & 6 others (1988). Differential expression of Insulin-like growth factor II mRNA in human primary liver cancers, benign liver tumours and liver cirrhosis. *Cancer Res.*, **48**, 6844.

COPEMAN, M.C. & HARRIS, H.J. (1990). The extracellular matrix of hybrids between melanoma cells and normal fibroblasts. *J. Cell Sci.*, **91**, 281.

COULOMBE, B. & SKUP, D. (1986). Expression of a synthetic human interferon a1 gene with modified sequences in mammalian cells. *Gene*, **46**, 89.

DAUGHADAY, W.H., EMANUELE, M.A., BROOKS, M.H., BARBATO, A.L., KAPADIA, M.S. & ROTWEIN, P. (1988). Synthesis and secretion of IGF-II by a leiomyosarcoma with associated hypoglycaemia. *New Engl. J. Med.*, **305**, 1432.

DE PAGTER-HOLTHUIZEN, P., JANSEN, M., VAN SCHAIK, F.M.A. & 4 others (1987). The human insulin-like growth factor II gene contains two development specific promoters. *FEBS Letts*, **214**, 259.

GESSLER, M., POUSTKA, A.-M., CAVENEE, W., NEVE, R.L., ORKIN, S.H. & BRUNS, G.A.P. (1990). Homozygous deletions in Wilms' tumours of a zinc finger gene identified by chromosome jumping. *Nature*, **343**, 774.

GRAY, A., TAM, A.W., DULL, T.J. & 5 others (1987). Tissue specific and developmentally regulated transcription of the insulin-like growth factor II gene. *DNA*, **6**, 283.

HAN, V.K.M., D'ERCOLE, A.J. & LUND, P.K. (1987). Cellular localisation of somatomedin mRNA in the human fetus. *Science*, **236**, 193.

HAN, V.K.M., LUND, P.K., LEE, D.C. & D'ERCOLE, A.J. (1988). Expression of somatomedin mRNAs in the human fetus. *J. Clin. Endocrinol.Metab.*, **66**, 422.

HASELBACHER, G.K., IRMINGER, J.C., ZAPF, J., ZENGLER, W.H. & HUMBEL, R.E. (1987). Insulin-like growth factor in human adrenal pheochromocytomas and Wilms' tumours: expression of the mRNA and protein level. *Proc. Natl Acad. Sci. USA*, **84**, 1104.

HENRY, I., GRANDJOUAN, S., COULLIN, P. & 7 others (1989). Tumour specific loss of 11p15.5 alleles in del 11p13 Wilms' tumours and in familial adrenocortical carcinoma. *Proc. Natl Acad. Sci. USA*, **86**, 3247.

HILL, D.J. (1990). Relative abundance and molecular size of insulin-like growth factors I and II in human fetal tissues. *Early Human Development* (in press).

HILL, D.J., CAMACHO-HUBNER, C., RASHID, P., STRAIN, A.J. & CLEMMONS, D.R. (1989). Insulin like growth factor binding protein release by human fetal fibroblasts; dependancy on cell density and IGF peptides. *J. Mol. Endocrinol.*, **2**, 31.

HILL, D.J., CRACE, C.J., STRAIN, A.J. & MILNER, R.D.G. (1986). Regulation of amino acid uptake and deoxyribonucleic acid synthesis in isolated human fetal fibroblasts and myoblasts: effects of human placental lactogen, somatomedin-C, multiplication-stimulating activity and insulin. *J. Clin Endocrinol. Metab.*, **62**, 753.

HILL, D.J., STRAIN, A. & MILNER, R.D.G. (1987). Growth factors in embryogenesis. In *Oxford Reviews in Reproductive Biology*, Clarke, J. (ed.), pp. 147–169. OUP: Oxford.

HOPPENER, J.W.M., MOSSELMAN, S., ROHALL, P.J.M. & 6 others (1988). Expression of insulin-like growth factor I and II genes in human smooth muscle tumours. *EMBO J.*, **7**, 1379.

IRMINGER, J.-C., SCHOENLE, E.J., BRINER, J. & HUMBEL, R.E. (1989). Structural alteration of the IGF-II gene in Wilms tumours. *Eur. J. Pediatr.*, **148**, 620.

KOUFOS, A., HANSEN, M.F., COPELAND, N.E., JENKINS, N.A., LAMPKIN, B.C. & CAVENEE, W.K. (1985). Loss of heterozygosity in three embryonal tumours suggests a common pathogenetic mechanism. *Nature*, **316**, 330.

LEHRACH, H., DIAMOND, D., WOZNEY, J.M. & BOETKER, H. (1977). RNA molecular weight determination by gel electrophoresis under denaturing conditions; a critical reexamination. *Biochemistry*, **16**, 4743.

LITTLE, M.H., ABLETT, G. & SMITH, P.J. (1987). Enhanced expression of IGF-II is not necessary for Wilm's tumour progression. *Carcinogenesis*, **8**, 865.

MAITLAND, N.J., BROWN, K.W., POIRIER, V., SHAW, A.P.W. & WILLIAMS, J. (1989). Molecular and cell biology of Wilms' tumour. *Anti-Cancer Res.*, **9**, 1417.

MANN, R., MULLIGAN, R.C. & BALTIMORE, D. (1983). Construction of a retrovirus packaging mutant and its use to produce helper free defective retrovirus. *Cell*, **33**, 153.

MASSAGUE, J., KELLY, B. & MOTTOLA, C. (1985). Stimulation by IGFs is required for cellular transformation by type $\beta$ TGF. *J. Biol. Chem.*, **260**, 4551.

MIEREAU, G.W., BECKWITH, B. & WEEKS, D.A. (1987). Ultrastructure and histogenesis of renal tumours of childhood; an overview. *Ultrastructural Pathol.*, **11**, 313.

PING, A.J., REEVE, A.E., LAW, D.J., YOUNG, M.R., BOEHNKE, S. & FEINBERG, A.P. (1989). Genetic linkage of Beckwith Weidemann syndrome to 11p15. *Am. J. Hum. Genet.*, **44**, 720.

### References

REEVE, A.H., SHIH, S.A., RAIZIS, A.M. & FEINBERG, A.P. (1989). Loss of allelic heterozygosity at a second locus on chromosome 11 in sporadic Wilms' tumor cells. *Mol. & Cell Biol.*, **9**, 1799.

ROSE, E.A., GLASER, T., JONES, C. & 6 others (1990). Complete physical map of the WAGR region of 11p13 localises a candidate Wilms' tumour gene. *Cell*, **60**, 495.

RUBIN, H. (1988). Uniqueness of each spontaneous transformant for a clone of Balbc 3T3 fibroblasts. *Cancer Res.*, **48**, 2512.

SCHOFIELD, P.N. (1991) Developmental tumours in 'The Cancer Cell' eds. Sirova, K., Evan, G., and Watson, J.V. *British Medical Bulletin*, **47**, 227.

SCHOFIELD, P.N. & TATE, V.E. (1987). Regulation of human IGF-II transcription in human fetal and adult tissues. *Development*, **101**, 793.

SCHOFIELD, P.N., TURNER, R.C., CONNOR, H. & ZAPF, J. (1989). Tumour hypoglycaemia: raised human IGF-II mRNA associated with reduced plasma somatomedin. *Br. J. Cancer*, **60**, 661.

SCOTT, J., COWELL, J., ROBERTSON, M.E. & 8 others (1985). Insulin-like growth factor II gene expression in Wilms' tumour and embryonic tissues. *Nature*, **317**, 260.

SU, T.-S., LIU, W.-Y., HAN, S.-H. & 4 others (1989). Transcripts of Insulin like growth factors I and II in human hepatoma. *Cancer Res.*, **49**, 1773.

SUSSENBACH, J.S. (1989). The gene structure of the insulin-like growth factor family. *Prog. Growth Factor Res.*, **1**, 33.

TEPPER, R.I., PATTENDALE, P.K. & LEDER, P. (1988). Murine IL4 displays potent anti tumour activity *in vivo*. *Cell*, **57**, 503.

TODARO, G. & GREEN, H.J. (1963). Quantitative studies of the growth of mouse embryo cells in culture and their establishment. *J. Cell Biol.*, **17**, 299.

TRICOLI, J.V., RALL, L.B., KARAKOUSIS, C.F. & 4 others (1986). Enhanced levels of insulin like growth factor messenger RNA in human colon carcinomas and liposarcomas. *Cancer Res.*, **46**, 6169.

WHARTON, W., VAN WYK, J.J. & PLEDGER, W.J. (1981). Inhibition of Balb/c 3T3 cells in late G1; commitment to DNA synthesis controlled by somatomedin C. *J. Cell Physiol.*, **107**, 31.

WHITELAW, E., HOGBEN, P., HANSCOMBE, O. & PROUDFOOT, N.J. (1989). Transcriptional promiscuity of the human α1 globin gene. *Mol. & Cell Biol.*, **9**, 241.

WILKINS, R.J., MOOLENAAR, A.J., OHLSSON, R., REEVE, A.E., YUN, K. & BECROFT, D.M.O. (1989). Wilms' tumourigenesis: insulin-like growth factor II expression and blocked differentiation. In *Cancer Cells* pp. 321–326. Cold Spring Harbour Laboratory: New York.